# Establishment of a method for determining the origin of glutamic acid in processed food based on carbon and nitrogen stable isotope ratios

**DOI:** 10.1016/j.heliyon.2019.e01169

**Published:** 2019-01-25

**Authors:** Kazuhiro Kobayashi, Yoichi Yatsukawa, Masaharu Tanaka, Soichi Tanabe, Mitsuru Tanaka, Takuya Suzuki

**Affiliations:** a Global Food Safety Institute, Nissin Foods Holdings Co., Ltd., 2100 Tobuki-machi, Hachioji, Tokyo 192–0001, Japan; b Global Innovation Research Center, Nissin Foods Holdings Co., Ltd., 2100 Tobuki-machi, Hachioji, Tokyo 192–0001, Japan; c Graduate School of Biosphere Science, Hiroshima Univ., Kagamiyama, Higashihiroshima, Hiroshima 739-8528, Japan

**Keywords:** Food analysis, Food science

## Abstract

We developed a discriminant method based on the stable isotope ratio of carbon and nitrogen (δ^13^C and δ^15^N) to evaluate whether monosodium glutamate (MSG) is used in processed food samples. δ^13^C measurements were performed by elemental analyzer/isotope-ratio mass spectrometry (EA/IRMS) for on glutamic acid isolated from samples at high purity, and δ^15^N measurements were performed by gas chromatography/combustion/IRMS (GC/C/IRMS) following the purification and derivatization steps. By applying these methods, the δ^13^C and δ^15^N values for glutamic acid present in a wide variety of processed foods were obtained. Subsequently, discriminant analysis, which is a statistical analysis method, was performed by using the δ^13^C and δ^15^N values from seasoning MSG and glutamic acid from foodstuffs of known origin, and the discriminant function was derived. By substituting the measured δ^13^C and δ^15^N values of processed food samples into this discriminant function and classifying samples into two groups, seasoning MSG (the seasoning group) and glutamic acid in foodstuffs (the foodstuff group), we determined whether seasoning MSG had been used in the processed food samples. As a result, the accuracy of distinguishing between the seasoning group and the foodstuff group was very high, i.e., 96.2%, indicating that the proposed method is a highly robust and accurate method for determining whether seasoning MSG has been used in for processed foods.

## 1 Introduction

Monosodium glutamate (MSG) is the sodium salt of glutamic acid and is used worldwide as a typical umami seasoning. Glutamic acid is naturally present in many foodstuffs, including kelp, tomatoes, and cheese. On the other hand, the majority of MSG that is used as a seasoning is industrially produced by the fermentation of sugar cane molasses, corn, cassava, tapioca starch, among others [1, 2].

In recent years, there has been a consumer trend to avoid the intake of added MSG and an increased desire for natural-tasting food [3]. Indeed, in countries such as the United States, many processed foods bear the indication “No MSG” or “MSG free”. However, these processed foods also contain glutamic acid derived from foodstuffs, and it is difficult to distinguish glutamic acid from seasoning MSG [4]. Therefore, the development of technology to determine whether MSG is used as a seasoning in commercially available processed foods could provide highly reliable information to consumers, which is of particular interest for the current trend.

Thus, to establish a method for distinguishing whether the glutamic acid present in food products is added seasoning MSG or natural glutamic acid, we utilized isotope-ratio mass spectrometry (IRMS), as reported in our previous publications [5, 6]. IRMS has often been used in ecology and the geosciences to precisely measure the stable isotope ratios of hydrogen, carbon, nitrogen, oxygen and sulfur [7, 8]. The stable isotope ratios of these elements are also useful for determining the origin and authenticity of foodstuffs in the field of food science [9, 10, 11, 12]. This approach is based on the principle that these isotope ratios in foodstuffs primarily reflect those that are either fixed or consumed by the organism from which the product is derived. In plants, the isotopic compositions of carbon and nitrogen (δ^13^C and δ^15^N) reflect those of carbon dioxide fixed via photosynthesis and of nitrogenous compounds in soil, respectively [13, 14]. For example, δ^13^C of C4 plants, such as corn and sugarcane, significantly differs from that of C3 plants, including most crops, such as rice and wheat (C4: −16‰ to −9‰ vs. C3: −34‰ to −24‰) [15]. δ^15^N is also used for estimating trophic levels, and δ^15^N of organisms with high trophic levels has high values because ^15^N enrichment during trophic transfer integrates a number of biochemical processes that accompany isotopic fractionation during nitrogen metabolism [16, 17]. Therefore, it should be possible to differentiate the origin of specimens based on isotopic composition differences.

Initially, we developed a novel method to isolate glutamic acid from food samples and to subsequently measure δ^13^C via elemental analyzer/isotope-ratio mass spectrometry (EA/IRMS) [5]. In our subsequent report [6], we applied the previously reported method for measuring δ^15^N in amino acids [18] to determine the δ^13^C and δ^15^N values of glutamic acid with known origins in seasoning MSG (6 samples from C3 plants and 31 samples from C4 plants) and in various foodstuffs (53 samples). Using the obtained results, we performed a discriminant analysis, called quadratic discriminant analysis (QDA), to classify glutamic acid according to its origin with a high success rate (accuracy of 96.7%).

It has also been reported that differences exist in the δ^13^C and δ^15^N values for seasoning MSG and for glutamic acid in foodstuffs, and discriminant analysis has been shown to be able to distinguish between the two [6]. Thus, we used of discriminant analysis to determine whether the glutamic acid present in commercially available processed food is glutamic acid from seasoning MSG or is inherently present in foodstuffs. Ultimately, we aimed to determine whether seasoning MSG was used in processed food samples.

## 2 Materials and methods

### 2.1 Samples

As representative examples of processed foods, we purchased instant noodles (19 samples), instant soups (7 samples), potato chips (5 samples), retort curry (4 samples), cheese (3 samples), salad dressing (2 samples), hamburger patties (2 samples), chicken nuggets (2 samples), ham (2 samples), sausages (2 samples), bacon (2 samples) and canned fish (2 samples) in which it was obvious whether seasoning MSG was added from the declaration on the packages (e.g., “No MSG” and “MSG free”) or the ingredient list (Table 1(A)). For each sample, the glutamic acid content was measured using the photometric ninhydrin method [19] (Table 1(B)).

**Table 1:** List of basic information, stable isotope ratios and discrimination results for the processed foods used in this study.

(A)				(B)					(C)	
No.	Samples	Country of manufacture	Addition of seasoning MSG	Glutamic acid content [g/100 g]	δ^13^C [‰]_VPDB_^a^		δ^15^N [‰]_AIR_^a^		Three-group discrimination analysis^b^^,^^d^	Two-group discrimination analysis^c^^,^^d^
					Mean	SD	Mean	SD		
1	Instant noodles No. 1	Japan	Yes	1.00	−15.9	0.1	−4.7	0.3	C4 plant-derived seasoning	Seasoning
2	Instant noodles No. 2	Japan	Yes	1.17	−14.4	0.1	−6.8	0.3	C4 plant-derived seasoning	Seasoning
3	Instant noodles No. 3	Japan	Yes	1.33	−16.9	0.1	−4.7	0.4	C4 plant-derived seasoning	Seasoning
4	Instant noodles No. 4	United States	Yes	0.67	−15.8	<0.1	−5.0	0.1	C4 plant-derived seasoning	Seasoning
5	Instant noodles No. 5	United States	Yes	0.87	−15.1	0.1	−5.7	0.2	C4 plant-derived seasoning	Seasoning
6	Instant noodles No. 6	United States	Yes	1.20	−14.9	0.1	−4.9	0.1	C4 plant-derived seasoning	Seasoning
7	Instant noodles No. 7	Japan	No	0.07	−17.9	0.1	−0.8	0.5	Foodstuff	Foodstuff
8	Instant noodles No. 8	Japan	No	0.06	−22.4	<0.1	−0.8	0.1	Foodstuff	Foodstuff
9	Instant noodles No. 9	Japan	No	0.23	−24.3	<0.1	0.3	0.3	Foodstuff	Foodstuff
10	Instant noodles No. 10	Japan	No	0.13	−21.6	<0.1	4.7	0.4	Foodstuff	Foodstuff
11	Instant noodles No. 11	Japan	No	0.04	−17.0	0.1	13.0	0.6	Foodstuff	Foodstuff
12	Instant noodles No. 12	United States	No	0.18	−14.3	0.1	0.7	0.4	Foodstuff	Foodstuff
13	Instant noodles No. 13	United States	No	0.19	−18.3	0.1	−1.9	0.2	Foodstuff	Foodstuff
14	Instant noodles No. 14	United States	No	0.19	−17.7	0.3	−0.7	0.3	Foodstuff	Foodstuff
15	Instant noodles No.15	United States	No	0.05	−16.6	0.1	2.5	0.4	Foodstuff	Foodstuff
16	Instant noodles No. 16	United States	No	0.05	−25.3	0.1	0.6	0.2	Foodstuff	Foodstuff
17	Instant noodles No. 17	United States	No	0.10	−26.2	<0.1	1.7	0.3	Foodstuff	Foodstuff
18	Instant noodles No. 18	India	No	0.16	−22.9	<0.1	1.1	0.2	Foodstuff	Foodstuff
19	Instant noodles No. 19	India	No	0.02	−21.6	0.1	1.6	0.1	Foodstuff	Foodstuff
20	Instant soup No. 1	Japan	Yes	4.98	−13.1	<0.1	−6.7	0.4	C4 plant-derived seasoning	Seasoning
21	Instant soup No. 2	Japan	Yes	1.69	−13.0	<0.1	−6.9	0.1	C4 plant-derived seasoning	Seasoning
22	Instant soup No. 3	Japan	No	0.20	−13.6	0.1	−4.9	0.6	**C4 plant-derived seasoning**	**Seasoning**
23	Instant soup No. 4	Japan	No	0.21	−18.1	0.1	2.1	0.1	Foodstuff	Foodstuff
24	Instant soup No. 5	India	Yes	5.24	−27.8	0.1	−4.9	0.3	C3 plant-derived seasoning	Seasoning
25	Instant soup No. 6	Japan	No	0.24	−14.6	0.2	−2.6	0.1	Foodstuff	Foodstuff
26	Instant soup No. 7	Japan	No	0.60	−16.6	0.1	2.2	0.1	Foodstuff	Foodstuff
27	Potato chips No. 1	Japan	Yes	1.07	−17.6	0.0	−4.0	0.3	C4 plant-derived seasoning	Seasoning
28	Potato chips No. 2	Thailand	Yes	1.63	−26.4	0.1	−6.3	0.3	C3 plant-derived seasoning	Seasoning
29	Potato chips No. 3	Thailand	Yes	0.87	−16.2	0.1	−5.6	0.3	C4 plant-derived seasoning	Seasoning
30	Potato chips No. 4	Japan	No	0.13	−25.8	0.1	0.2	0.5	Foodstuff	Foodstuff
31	Potato chips No. 5	United States	No	0.24	−25.7	0.1	2.1	0.6	Foodstuff	Foodstuff
32	Retort pouched curry No. 1	Japan	Yes	0.61	−15.3	0.2	−2.9	0.5	C4 plant-derived seasoning	**Foodstuff**
33	Retort pouched curry No. 2	Japan	Yes	1.82	−22.8	<0.1	−3.0	0.3	**Foodstuff**	Seasoning
34	Retort pouched curry No. 3	Japan	No	0.05	−16.3	<0.1	3.8	0.2	Foodstuff	Foodstuff
35	Retort pouched curry No. 4	Japan	No	0.12	−23.4	<0.1	3.6	0.6	Foodstuff	Foodstuff
36	Cheese No. 1	Japan	Yes	0.11	−21.2	<0.1	−5.9	0.3	**Foodstuff**	Seasoning
37	Cheese No. 2	Japan	No	0.15	−15.2	0.1	4.0	0.2	Foodstuff	Foodstuff
38	Cheese No. 3	Japan	No	0.15	−18.1	0.1	5.2	0.1	Foodstuff	Foodstuff
39	Dressing No. 1	United States	Yes	0.27	−11.1	<0.1	−5.6	0.4	**Foodstuff**	Seasoning
40	Dressing No. 2	Japan	No	0.06	−19.9	<0.1	3.9	0.4	Foodstuff	Foodstuff
41	Hamburger steak No. 1	Japan	Yes	0.15	−17.4	0.3	−3.6	0.4	C4 plant-derived seasoning	Seasoning
42	Hamburger steak No. 2	Japan	No	0.04	−23.8	<0.1	2.9	0.2	Foodstuff	Foodstuff
43	Chicken nugget No. 1	Japan	Yes	0.19	−14.8	0.1	−4.6	0.2	C4 plant-derived seasoning	Seasoning
44	Chicken nugget No. 2	Japan	No	0.06	−17.7	0.1	3.3	0.4	Foodstuff	Foodstuff
45	Ham No. 1	Japan	Yes	0.26	−12.7	0.1	−6.6	0.4	C4 plant-derived seasoning	Seasoning
46	Ham No. 2	Spain	No	0.23	−18.5	<0.1	7.4	0.1	Foodstuff	Foodstuff
47	Sausage No. 1	Japan	Yes	0.22	−15.0	0.2	−4.8	0.4	C4 plant-derived seasoning	Seasoning
48	Sausage No. 2	Japan	No	0.25	−14.1	0.3	−3.0	0.4	Foodstuff	Foodstuff
49	Bacon No. 1	Japan	Yes	0.39	−12.8	<0.1	−5.4	0.4	C4 plant-derived seasoning	Seasoning
50	Bacon No. 2	Japan	No	0.03	−16.1	<0.1	2.7	0.4	Foodstuff	Foodstuff
51	Canned fish No. 1	Japan	Yes	0.30	−21.3	0.1	−2.6	0.4	**Foodstuff**	Seasoning
52	Canned fish No. 2	Japan	No	0.01	−17.3	<0.1	18.7	0.6	Foodstuff	Foodstuff

a N = 3.

b Results of the three-group discriminant analysis (C3 plant-derived seasoning group, C4 plant-derived seasoning group, Foodstuff group) based on functions 1 and 2.

c Results of the two-group discriminant analysis (Seasoning group, Foodstuff group) based on function 3.

d Samples that were erroneously classified are represented in bold.

### 2.2 Reagents and chemicals

Amino standards of ʟ-Alanine (δ^13^C vs. VPDB = −19.6‰ ± 0.2, δ^15^N vs. AIR = 26.1‰ ± 0.2), ʟ-aspartate (δ^13^C vs. VPDB = −23.95‰ ± 0.02, δ^15^N vs. AIR = 35.20‰ ± 0.05), two types of glycine (A, δ^13^C vs. VPDB = −32.3‰ ± 0.2, δ^15^N vs. AIR = 1.12‰ ± 0.2; B, δ^13^C vs. VPDB = −60.02‰ ± 0.02, δ^15^N vs. AIR = −26.63‰ ± 0.02), ʟ-histidine (δ^13^C vs. VPDB = −11.4‰ ± 0.2, δ^15^N vs. AIR = −7.6‰ ± 0.2), ʟ-phenylalanine (δ^13^C vs. VPDB = −11.20‰ ± 0.02, δ^15^N vs. AIR = 1.70‰ ± 0.06), ʟ-hydroxyproline (δ^13^C vs. VPDB = −12.66‰ ± 0.02, δ^15^N vs. AIR = −9.17‰ ± 0.07), and ʟ-norleucine (δ^13^C vs. VPDB = −28.85‰ ± 0.03, δ^15^N vs. AIR = 18.96‰ ± 0.11) were purchased from Shoko Scientific Corporation (Saitama, Japan) and used as working standards for the δ^13^C and δ^15^N analyses.

Distilled water (HPLC grade), hydrochloric acid (precision analysis grade), sodium hydroxide (special grade), n-hexane (pesticide residue and polychlorinated biphenyl analysis grade), dichloromethane (HPLC grade), methanol (HPLC grade), 2-propanol (HPLC grade), 25% ammonia water (special grade), ammonium hydrogen carbonate (first grade), activated carbon (for chromatography), thionyl chloride (Wako special grade), pivaloyl chloride (practical grade), anhydrous magnesium sulfate (practical grade), and ʟ-monosodium glutamate monohydrate (special grade; MSG reagent, δ^13^C = −14.7‰, δ^15^N = 5.6‰) were purchased from Wako Pure Chemical Industries (Osaka, Japan). ODS-A-HG (50 μm particle size, 12 nm pore size; YMC, Kyoto, Japan) was used as the octadecylsilylated silica gel (C18) column resin, and Amberlite^®^ IR120B (hydrogen form; Organo Corporation, Tokyo, Japan) and AG^®^ 50W-X8 (200–400 mesh, hydrogen form; Bio-Rad Laboratories, Hercules, CA, US) were used as strong cation exchange resins. Merck Millipore's (Darmstadt, Germany) Millex^®^-LH syringe-driven filter unit (hydrophilic PTFE membrane, 0.45 μm pore size) and an Ultrafree^®^-MC-GV centrifugal filter unit (PVDF membrane, 0.22 μm pore size) were used for filtration.

### 2.3 δ^13^C analysis of extracted glutamic acid

#### 2.3.1 Extraction of glutamic acid for δ^13^C analysis

To measure the δ^13^C values by EA/IRMS, glutamic acid was isolated from the samples according to our previous report [5] with a minor modification.

Samples were collected so that the amount of free glutamic acid would be ≥10 mg. Next, 30 mL of distilled water was added to each sample as well as 10 mL of 1 mol L^−1^ hydrochloric acid, 15 mL of n-hexane, and 10 mL of dichloromethane (in the case where the sampling amount was >10 g, the amount of each reagent was tripled), and the resulting mixture was shaken for 30 min. Following centrifugation (2,140 ×g, 3 min), the upper layer was removed, and the lower layer was loaded onto an activated carbon (3 g)/C18 (3 g) column preconditioned with methanol and distilled water (30 mL each, in this order), followed by an additional 10 mL of distilled water. The eluent was collected and loaded onto a column containing a strongly acidic cation exchange resin (100 g, Amberlite^®^ IR120B) preconditioned with distilled water, 1 mol L^−1^ hydrochloric acid, and distilled water (300 mL each, in this order). After washing with an additional 400 mL of distilled water, the column was eluted with 200 mL of an aqueous ammonia solution (10% v/v). The eluent was then concentrated to dryness using a rotary evaporator at 60 °C, and the residue was dissolved in 3 mL of 65% v/v methanol and filtered using a Millex^®^-LH syringe-driven filter unit. The filtrate was then subjected to preparative HPLC (conditions described in Section 2.3.2.). Then, following the isolation of the glutamic acid peak that eluted at approximately 27 min, the isolated portion was concentrated to dryness using a rotary evaporator at 60 °C. The glutamic acid crystals obtained were employed for the EA/IRMS measurements (conditions described in Section 2.3.3.).

#### 2.3.2 Isolation of glutamic acid by preparative HPLC

For the pretreatment for the δ^13^C measurements (in Section 2.3.1.), a preparative HPLC system manufactured by Shimadzu Corporation (Kyoto, Japan) was used to isolate glutamic acid from the eluate of a strongly acidic cation exchange column, which consisted of a controller (CBM-20A), a feed pump (LC-20AP), an autosampler (SIL-10AP), a UV detector (SPD-20A), and a fraction collector (FRC-10A). The preparative column and guard column included a Shodex Asahipak NH2P-90 20F (300 mm × 20 mm i.d., 9 μm particle size) and a Shodex Asahipak NH2P-130G 7B (50 mm × 7.5 mm i.d., 13 μm particle size), respectively (Showa Denko, Tokyo, Japan). A mixture of 100 mmol L^−1^ aqueous ammonium bicarbonate and methanol (35:65, v/v) was used as the mobile phase at a flow rate of 7 mL min^−1^. The column temperature was set at room temperature, and ultraviolet absorbance at 210 nm was used to detect the signal corresponding to glutamic acid. Since it was difficult to completely remove ammonium salt in the glutamic acid isolated by preparative HPLC, δ^15^N could not be simultaneously analyzed with δ^13^C by EA/IRMS.

#### 2.3.3 Analysis by EA/IRMS

The δ^13^C values were measured using an online EA/IRMS system manufactured by Thermo Fisher Scientific (Bremen, Germany) included a Flash 2000 electron analyzer, a ConFlo IV interface, and a Delta V Advantage isotope-ratio mass spectrometer. For EA, the temperatures of the oxidation furnace, the reduction furnace, and the column oven were set to be 1,000, 680, and 50 °C, respectively, whereas the flow rates of the carrier gas (helium) and the combustion gas (oxygen) were set to be 100 and 175 mL min^−1^, respectively. Approximately 0.3 mg each of the four standard reagents (ʟ-alanine, two types of glycine (A and B), and ʟ-histidine) and the samples were weighed into tin capsules, wrapped, and then used for the measurements.

### 2.4 δ^15^N analysis of extracted glutamic acid

#### 2.4.1 Extraction of glutamic acid for δ^15^N analysis

To measure the δ^15^N values by GC/C/IRMS, glutamic acid was extracted from the samples and derivatized according to the methods of Chikaraishi et al. [18] and our previous report [6] with a minor modification.

The samples were collected such that the amount of free glutamic acid was ≥0.2 mg. To each sample, 6 mL of distilled water, 2 mL of 1 mol L^−1^ hydrochloric acid, 3 mL of n-hexane, and 2 mL of dichloromethane were added, and the resulting mixture was shaken for 10 min. Following centrifugation (2,140 ×g, 3 min), the upper layer was removed, and the lower layer was loaded onto a strongly acidic cation exchange resin (10 mL, AG^®^50W-X8) preconditioned in the following order: distilled water, 1 mol L^−1^ hydrochloric acid, distilled water, 1 mol L^−1^ sodium hydroxide, distilled water, and 1 mol L^−1^ hydrochloric acid (15 mL each). After washing with 40 mL of distilled water, the column was eluted with 20 mL of 10% v/v aqueous ammonia solution. The eluent was concentrated to dryness using a rotary evaporator at 60 °C. Subsequently, 0.5 mL of thionyl chloride/2-propanol (4:1, v/v) was added to the residue and allowed to react for 2 h at 110 °C. After cooling to room temperature, the reaction solution was dried under flowing nitrogen gas, and then 0.1 mL of pivaloyl chloride and 0.4 mL of dichloromethane were added and allowed to react for 2 h at 110 °C. After cooling to room temperature, the reaction solution was dried under flowing nitrogen gas, and then 0.3 mL of distilled water, 0.3 mL of n-hexane, and 0.2 mL of dichloromethane were added prior to shaking. The top layer was filtered through an Ultrafree^®^-MC-GV centrifugal filter unit packed with anhydrous magnesium sulfate. This operation was repeated three times. The obtained filtrate was dried under flowing nitrogen gas and dissolved in 0.5 mL of dichloromethane. The resulting solution was subjected to GC/IRMS measurements (conditions described in Section 2.4.2.).

#### 2.4.2 Analysis by GC/C/IRMS

The δ^15^N values were measured using an online GC/C/IRMS system manufactured by Thermo Fisher Scientific, which included the GC apparatus (Trace 1310), a GC Isolink II, a ConFlo IV interface, and a Delta V Advantage isotope-ratio mass spectrometer. An Ultra 2 column (50 m × 0.32 mm i.d., 0.54 μm film; Agilent Technologies, Santa Clara, CA, US) was used as the GC column, and the temperature of the column oven was set to 40 °C initially (2.5 min hold) and then increased at 20 °C min^−1^ to 110 °C (0 min hold), 3.2 °C min^−1^ to 150 °C (0 min hold), 9 °C min^−1^ to 220 °C (10 min hold), and 30 °C min^−1^ to 250 °C (5 min hold). The temperature at the inlet was 270 °C, the injection dose was 1 μL (split-less), the flow rate was 1.4 mL min^−1^, and the temperature in the reactor was 1,000 °C. The amino acid standard was prepared by weighing and mixing each standard reagent (1 mg, ʟ-aspartate, glycine (B), ʟ-phenylalanine, ʟ-hydroxyproline, and ʟ-norleucine), followed by derivatization as described in Section 2.4.1.

### 2.5 Stable carbon and nitrogen isotope ratio analyses

The carbon and nitrogen isotopic compositions are expressed with conventional δ notation against the international substance [Vienna PeeDee Belemnite (VPDB) for δ^13^C and atmospheric nitrogen (AIR) for δ^15^N] [20]:

δiE = [(iR_Sample_/iR_Standard_)/iR_Standard_] × 1000

where i is the mass number of the heavier isotope of element E (^13^C and ^15^N), R_Sample_ is the isotope ratio of a sample (^13^C/^12^C and ^15^N/^14^N), and R_Standard_ is the isotope ratio of the international substance.

Four δ^13^C-known amino acids standards (ʟ-alanine, two types of glycine (A and B), and ʟ-histidine) used for a calibration curve were analyzed every 4 or 5 samples to confirm the reproducibility of the isotope measurement by EA/IRMS. Analytical errors (1σ) of the standards were better than 0.1‰ with a minimum sample amount of 100 μg C. Standard mixtures of 5 δ^15^N-known amino acids (ʟ-aspartate, glycine (B), ʟ-phenylalanine, ʟ-hydroxyproline, and ʟ-norleucine) used for a calibration curve were analyzed every 4 or 5 samples to confirm the reproducibility of the isotope measurement by GC/C/IRMS. Analytical errors (1σ) of the standards were better than 0.5‰ with a minimum sample amount of 800 ng N. The δ^13^C and δ^15^N of glutamic acid was determined using this method.

The δ^13^C and δ^15^N of glutamic acid in the processed food were repeatedly measured (3 or 5 times) on the same day. All results are represented as the average ± standard deviations (SD). The mean standard deviation (1σ) of the isotopic values of glutamic acid were better than ±0.3‰ for the δ^13^C measurements by EA/IRMS and were better than ±0.6‰ for the δ^15^N measurements by GC/C/IRMS.

### 2.6 Discriminant analysis

A discriminant analysis was performed using JMP 14.0.0 software (SAS Institute, Cary, NC, US) [9, 21]. Based on the δ^13^C and δ^15^N values of the C3 plant-derived seasoning MSG (6 samples), the C4 plant-derived seasoning MSG (31 samples) and the various foodstuffs (C3 plants, such as soy bean and tomato, and C4 plants, such as corn and kelp, marine products, including shrimp and tuna, mushrooms, and livestock products, including beef and chicken; a total of 53 samples) whose origins were already known [6], discriminant analyses were performed between the three groups (the C3 plant-derived seasoning group, the C4 plant-derived seasoning group, and the foodstuff group) and between the two groups (the seasoning group and the foodstuff group).

## 3 Results and discussion

### 3.1 Confirmation of isotopic fractionation via pretreatment of processed food samples

In our previous reports [5, 6], the samples were first subjected to acid hydrolysis to obtain glutamic acid-constituting proteins. However, in this study, we aimed to measure the δ^13^C and δ^15^N values only for free glutamic acid; thus, acid hydrolysis was not performed. Furthermore, to obtain a sufficient quantity of glutamic acid for the measurements, it was necessary to increase the sample quantities used for those with low glutamic acid contents, and the quantity of reagent used during extraction was also increased to ensure good operability.

First, we confirmed that isotopic fractionation does not occur during the pretreatment methods. To minimize the influence of the glutamic acid derived from the sample, an MSG reagent (δ^13^C = −14.7‰, δ^15^N = 5.6‰ by EA/IRMS) was added in excess to the processed food samples with relatively low glutamic acid contents so that the proportion of glutamic acid originating from the MSG reagent was ≥95%. As representative processed food samples, we used instant noodles as high in carbohydrates, fats, and sodium; potato chips as high in carbohydrates and fats; salad dressings as high in fats and sodium; and bacon as high in fats and protein. These samples to which the MSG reagent was added were subjected to each pretreatment for δ^13^C and δ^15^N measurements prior to measurement of their stable isotope ratios (Table 2). The results of the δ^13^C measurements gave a standard deviation for N = 5 that was <0.1‰, and the difference relative to the δ^13^C value of the added MSG reagent (at −14.7‰) was between −0.4 and 0.3‰. Furthermore, the results of the δ^15^N measurements were such that the standard deviation for N = 5 was <0.4‰, and the difference relative to the δ^15^N value of the added MSG reagent (at −5.6‰) was between −0.3 and 0.5‰. Thus, because the isotope ratio deviation determined via the pretreatment methods and subsequent measurements was within ±0.5‰ for both δ^13^C and δ^15^N, the pretreatment methods for the processed food samples resulted in almost no isotopic fractionation. We therefore considered the pretreatment methods to be sufficiently accurate to fulfill the objectives of this study.

**Table 2:** Confirmation of isotopic fractionation in the measurement of the δ^13^C and δ^15^N values of glutamic acid in the processed foods.

Samples	Main ingredients [weight per 100 g]^a^	Measurement method of δ^13^C [‰]_VPDB_^b^			Measurement method of δ^15^N [‰]_AIR_^b^		
		Mean	SD	Difference from MSG reagent	Mean	SD	Difference from MSG reagent
MSG reagent	–	−14.7	–	–	−5.6	–	–
Instant noodles	Protein (9.0 g) **Fat (19.0 g)** **Carbohydrate (62.4 g)** **Sodium (1,451 mg)**	−15.1	0.1	−0.4	−5.1	0.1	0.5
Potato chips	Protein (4.7 g) **Fat (33.3 g)** **Carbohydrate (55.7 g)** Sodium (197 mg)	−14.9	0.1	−0.2	−5.3	0.3	0.3
Dressing	Protein (2.7 g) **Fat (47.3 g)** Carbohydrate (7.3 g) **Sodium (1,280 mg)**	−14.7	0.1	0.0	−5.3	0.4	0.3
Bacon	**Protein (14.5 g)** **Fat (28.3 g)** Carbohydrate (2.4 g) Sodium (472 mg)	−14.4	0.0	0.3	−5.9	0.4	−0.3

a Value indicated in the nutritional ingredients list.

b N = 5.

### 3.2 Application of discriminant analysis to the processed food samples

In our previous study [6], we statistically analyzed the stable isotope ratio data of C3 plant-derived seasoning MSG (6 samples), C4 plant-derived seasoning MSG (31 samples), and glutamic acid contained in various foodstuffs (C3 plants, C4 plants, kelp, marine products, mushrooms, livestock products; a total of 53 samples) via canonical discriminant analysis; thus, it was possible to discriminate between C3 plant-derived seasoning MSG, C4 plant-derived seasoning MSG, and glutamic acid in the various foodstuffs with an accuracy of 96.7%. The discriminant functions obtained in this way are as follows:

Function 1: 0.154 [δ^15^N] + 0.141 [δ^13^C] − 2.744,

Function 2: 0.165 [δ^15^N] + 0.073 [δ^13^C] + 2.688.

Therefore, we evaluated whether discriminant analysis methods based on the use of these discriminant functions could be applied directly to processed foods that have undergone processes such as heating and drying.

**Fig. 1:**
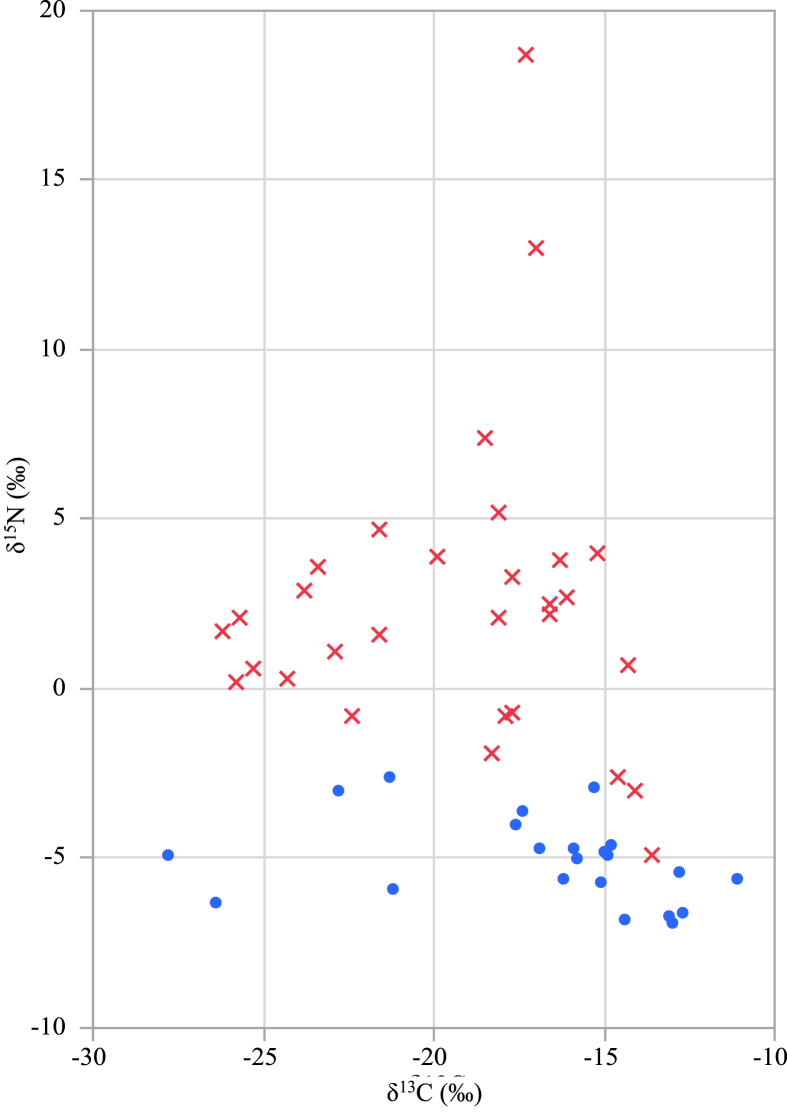
Two-dimensional plot of the δ^13^C and δ^15^N values of glutamic acid contained in processed foods. Blue “●” and red “×” show samples to which seasoning MSG was added and samples to which seasoning MSG was not added, respectively.

Initially, we determined the δ^13^C and δ^15^N values of glutamic acid present in a total of 52 processed food samples for which the use/nonuse of seasoning MSG (where the C3 or C4 plant derivation was unknown) was known from the declaration on the packages (e.g., “No MSG” and “MSG free”) or the ingredient list. The results are summarized in Table 1(B), and the corresponding two-dimensional plot of δ^13^C and δ^15^N is provided in Fig. 1.

The processed foods samples in which seasoning MSG was used had δ^13^C in the range of –27.8 to –11.1‰ and δ^15^N in the range of –6.9 to –2.6‰, and most samples were within the range of the C3 plant-derived seasonings MSG (δ^13^C: –29.1 to –26.1‰, δ^15^N: –6.6 to –2.5‰, from the previous report data [6]) or C4 plant-derived seasoning MSG (δ^13^C: –16.7 to –10.1‰, δ^15^N: –9.9 to –3.9‰, from the previous report data [6]). Additionally, the processed foods samples in which seasoning MSG was not used had δ^13^C in the range of –26.2 to –13.6‰ and δ^15^N in the range of –4.9 to 18.7‰, and most samples were within the range of the various foodstuffs (δ^13^C: –27.3 to –9.6‰, δ^15^N: –3.2 to +29.0‰, from the previous report data [6]).

The δ^13^C values of the processed food samples in which seasoning MSG was used reflected the isotopic compositions of the raw materials used for seasoning MSG production (e.g., sugar cane molasses and corn of the C4 plant, cassava and tapioca starch of the C3 plant), and the processed foods samples in which seasoning MSG was not used reflected the isotopic compositions of the main ingredients or food additives, such as hydrolyzed protein of meat or vegetables.

Compared with the δ^15^N values from the processed foods samples in which seasoning MSG was not used, those from the processed foods samples in which seasoning MSG was used were slightly lower isotopically. When seasoning MSG is produced via enzymatic microbial fermentation, the common nitrogen source is usually ammonia gas in solution [2, 22]. Because the isotope ratio of gaseous nitrogen in the ammonia raw material is 0‰, that of seasoning MSG is also expected to be 0‰. The reason why seasoning MSG exhibited slightly lower nitrogen isotope ratios was likely because of the isotopic fractionation of nitrogen that occurs during the MSG purification processes during manufacturing, such as decolorization and recrystallization [6].

**Fig. 2:**
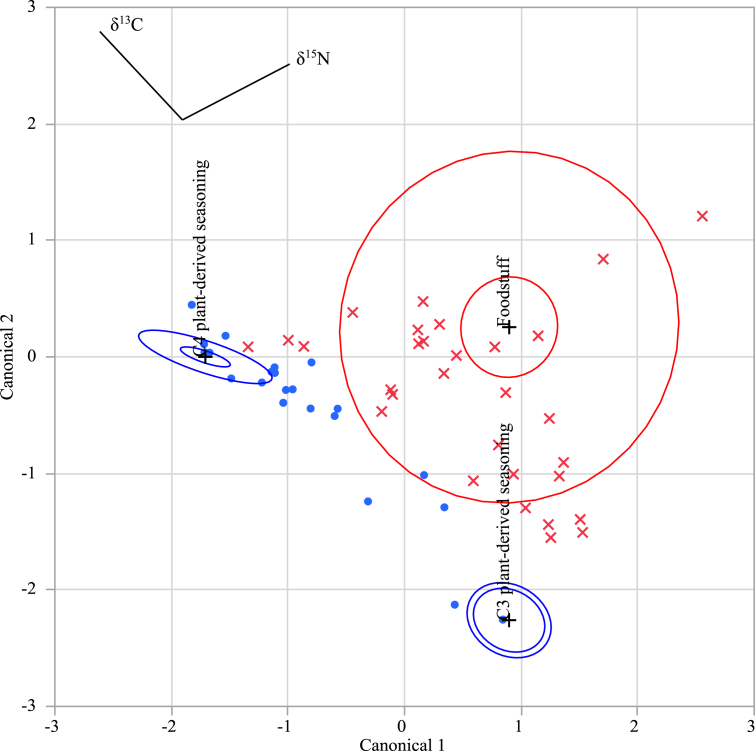
Two-dimensional plot of the discriminant scores calculated using discriminant functions 1 and 2. The discriminant scores obtained using functions 1 and 2 are shown on the horizontal and vertical axes, respectively. Blue “●” and red “×” show samples to which seasoning MSG was added and samples to which seasoning MSG was not added, respectively. The other symbols in the figure show the information obtained by discriminant analysis for the three groups (the C3 plant-derived seasoning group, the C4 plant-derived seasoning group, and the foodstuff group) using statistical analysis software for the δ^13^C and δ^15^N values of the 90 samples discussed in a previous report [6]. The inner ellipses indicate the 95% confidence interval for each group mean, and the outer ellipses indicate the region estimated to contain 50% of the population of each group. The set of rays indicates the degree of association for a covariate with the two variables. The coordinate points are denoted by “+” signs.

We then substituted these δ^13^C and δ^15^N results into the discriminant functions 1and 2 shown above, calculated the discriminant score, and determined to which group each sample could be classified (Table 1(C)). A two-dimensional plot of the discriminant scores is shown in Fig. 2. As a result, out of the 22 samples where seasoning MSG had been added, 16 samples were found to belong to the C4 plant-derived seasoning group, 2 samples belonged to the C3 plant-derived seasoning group, and the remaining 4 samples belonged to the foodstuff group. Furthermore, out of the 30 samples without any added seasoning MSG, 29 samples belonged to the foodstuff group while the remaining sample belonged to the C4 plant-derived seasoning group. In total, 47 of the 52 samples were classified with an accuracy of 90.4%.

The erroneous classification of five of the samples could be attributed to two possible factors; one is the effect of free glutamic acid in the raw materials, and the other is seasoning MSG contamination. First, in addition to seasoning MSG, the raw materials of the processed foods can also contain free glutamic acid, which could then influence the δ^13^C and δ^15^N values. A remarkable example would be the canned fish (tuna) of sample No. 51. Although seasoning MSG had been added, this sample was erroneously classified in the foodstuff group instead of the two seasoning groups. In general, tuna contains ∼1–10 mg/100 g of free glutamic acid [23], and large fish such as tuna are known to have high δ^15^N values (≥20‰) [7, 24]. Based on such facts, the free glutamic acid derived from the tuna may have shifted the δ^15^N value of the added seasoning MSG itself to the plus side. The second possible explanation could be unintended seasoning MSG contamination during the manufacturing process. Indeed, there are cases (e.g., sample No. 22, instant soup) for which samples were classified into the seasoning group due to the influence of MSG added to the raw materials used to prepare the processed food, despite the fact that no seasoning MSG had been directly added by the final manufacturer.

### 3.3 Determination of the presence or absence of added MSG via discriminant analysis between the seasoning group and the foodstuff group

The discriminant analysis in Section 3.2. classified the samples into three groups of glutamic acid origin, namely, the C3 plant-derived seasoning group, the C4 plant-derived seasoning group, and the foodstuff group; thus, so that the originating plant of the seasoning MSG (i.e., the C3 or C4 plant) could be distinguished. However, in practice, it is particularly desirable to determine whether seasoning MSG has been used, and the priority is to discriminate it with as high accuracy as possible. We therefore combined the C3 plant-derived seasoning group and the C4 plant-derived seasoning group into a single group (i.e., the seasoning group) and attempted to perform a two-group discriminant analysis.

For this purpose, the discrimination analysis between the two groups was performed using the δ^13^C and δ^15^N values from seasoning MSG and glutamic acid in foodstuffs of known origin that were acquired in our previous report [6], and the discriminant function obtained was as follows:

Function 3: 0.153 [δ^15^N] – 0.057 [δ^13^C] – 1.125.

**Fig. 3:**
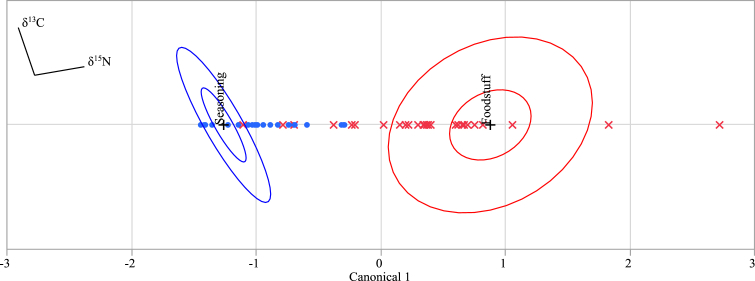
Plot of the discriminant scores calculated using discriminant function 3. Blue “●” and red “×” show samples to which seasoning MSG was added and samples to which seasoning MSG was not added, respectively. The other symbols in the figure show the information obtained by discriminant analysis using statistical analysis software between the two groups (the seasoning group and the foodstuff group) for the δ^13^C and δ^15^N values of the 90 samples discussed in a previous report [6]. The inner ellipses indicate the 95% confidence interval for each group mean, and the outer ellipses indicate the region estimated to contain 50% of the population of each group. The set of rays indicates the degree of association for a covariate with the two variables. The coordinate points are denoted by “+” signs.

Subsequently, we substituted the δ^13^C and δ^15^N values obtained for the glutamic acid present in the processed foods into discriminant function 3, determined the discriminant score (see Fig. 3), and analyzed to which group each sample belonged (Table 1(C)). As a result, 21 out of the 22 samples containing added seasoning MSG were classified into the seasoning group, while the remaining sample was classified into the foodstuff group. Furthermore, 29 out of 30 samples without any added MSG were classified into the foodstuff group, and the remaining sample was classified into the seasoning group. In total, 50 out of 52 samples were classified correctly with an accuracy of 96.2%, which was a superior result compared with that obtained with the method described in Section 3.2 (accuracy of 90.4%). The seasoning group region on the discriminant score plot, which was relatively small compared with the region for the foodstuff group (Fig. 2), increased when the C3 and C4 plant-derived seasoning groups were combined into a single seasoning group (Fig. 3). As a result, the sample incorrectly classified as the foodstuff group (due to its position close to the boundary of each group in the discriminant score plot) was considered to be correctly classified here as the seasoning group.

Based on the above results, it is apparent that this discriminant analysis method could determine with high accuracy whether the glutamic acid present in a given processed food originated from seasoning MSG or from the foodstuff itself, thereby confirming whether seasoning MSG had been added to the product.

Finally, as a future task for efficient operation, it is necessary to develop a simultaneous analytical method for δ^13^C and δ^15^N by either EA/IRMS or GC/C/IRMS. In short, the analytical methods could have been improved either by (1) optimizing the removal of ammonium salt from the mobile phase or finding an alternative mobile phase and analyzing both ^13^C and ^15^N via EA/IRMS or, (2) by using a derivatization technique with lower exogenous carbon to allow for ^13^C and ^15^N analysis via GC/C/IRMS. Furthermore, we can establish discrimination methods for other seasonings such as nucleic acids and organic acids using similar techniques.

## 4 Conclusions

We successfully developed a method to determine whether MSG is present in processed foods. To measure the δ^13^C and δ^15^N values of glutamic acid in processed food samples, glutamic acid was isolated at high purity and subjected to EA/IRMS for δ^13^C measurements, while δ^15^N measurements were performed by GC/C/IRMS after purification and derivatization. When discriminant analysis was performed based on the obtained δ^13^C and δ^15^N values of glutamic acid in various processed foods, the accuracy of distinguishing between the seasoning group and the foodstuff group was very high, i.e., 96.2%. We therefore conclude that the use of this method allows a scientific and objective evaluation of commercialized processed foods labeled as “No MSG” or “MSG free”, which in turn can confirm the reliability of such products and provide accurate information to consumers.

## Declarations

### Author contribution statement

Kazuhiro Kobayashi: Conceived and designed the experiments; Analyzed and interpreted the data; Contributed reagents, materials, analysis tools or data; Wrote the paper.

Yoichi Yatsukawa: Conceived and designed the experiments; Performed the experiments; Analyzed and interpreted the data; Contributed reagents, materials, analysis tools or data; Wrote the paper.

Masaharu Tanaka: Conceived and designed the experiments; Contributed reagents, materials, analysis tools or data; Wrote the paper.

Soichi Tanabe, Takuya Suzuki, Mitsuru Tanaka: Analyzed and interpreted the data; Wrote the paper.

### Funding statement

This research did not receive any specific grant from funding agencies in the public, commercial, or not-for-profit sectors.

### Competing interest statement

The authors declare no conflict of interest.

### Additional information

No additional information is available for this paper.
